# The enhanced interrogator: Dr. James Mitchell’s perspectives on enhanced interrogation

**DOI:** 10.1177/0957154X251358448

**Published:** 2025-08-10

**Authors:** Myles Balfe

**Affiliations:** 1 University College Cork, Ireland

**Keywords:** Enhanced Interrogation, torture, psychology, mental health, War on Terror

## Abstract

This article examines the historical narratives of one of the key psychologists who helped to develop and carry out the Enhanced Interrogation programme. The Enhanced Interrogation programme ran from the early to the mid-2000s. It used aggression to facilitate the acquisition of information from suspected terrorists. The programme was unique in its use of mental health professionals to design, implement and monitor its activities. The psychologist’s historical accounts were thematically analysed. The article argues that death anxiety and death guilt may drive some health professionals to support Enhanced Interrogation type activities. It also argues that health professionals may be unable to control deviance and violations in situations where Enhanced Interrogation activities are occurring.

## Introduction

On September 11th, 2001, the United States suffered a series of catastrophic attacks. Terrorists hijacked four passenger planes, flying two of them into the World Trade Center in New York. One plane crashed into the Pentagon, and the fourth, United Flight 93, crashed near Shanksville, Pennsylvania. Following the atrocities of that day, the United States launched a Global War on Terror. As part of this war, the United States tasked its foreign intelligence service, the CIA, to identify and disrupt future apocalyptic attacks.

The most controversial programme that the CIA initiated in the wake of 9/11 was known the “Enhanced Interrogation” programme. This programme used significant aggression to facilitate the extraction of information from suspected terrorists. In 2014, a U.S. Government Report (often referred to as “The Report” or “The Torture Report”) (SSIR, 2014) outlined the history and activities of the Enhanced Interrogation programme in considerable detail.

One of the most important revelations in The Report was the extent to which mental healthcare professionals were involved in Enhanced Interrogation. The Report zeroed in on the roles of two psychologists in the programme in particular. The real identities of these psychologists were later revealed to be Dr. James Mitchell and Dr. Bruce Jessen, former military psychologists. These two psychologists had important roles in assisting to develop, operationalize and monitor Enhanced Interrogation. They were referred to as “the architects of the most important interrogation program in the history of American counterterrorism” (Shane, 2009), amongst its “founding fathers” (Rosenberg, 2020a).

The international backlash by some against these two psychologists in the wake of The Report’s revelations was strong, with their involvement in the programme being viewed as professionally deviant (Leopold, 2014; Rosenberg, 2020b; Shane, 2009). One health professional association said that the two psychologists’ participation in the programme “represents one of the gravest breaches of medical ethics in U.S. history” (Physicians for Human Rights, 2020; see also Physicians for Human Rights, 2014). Although their Enhanced Interrogation activities were defended by some, others argued that their actions contributed to efforts that were futile, corroded values, and led to serious strategic mistakes (Stevenson, 2015; Johnson et al., 2016; Miles, 2020).

In the years following the release of The Report, Dr. Mitchell gave a number of detailed accounts about his perspectives on Enhanced Interrogation. His accounts were viewed sceptically by some audiences (Taguba and Cooper, 2017) but it has also been acknowledged that they provided insights that were “sometimes at odds with. . .previous portrayals” (Fink and Risen, 2017). Given the importance of the Enhanced Interrogation programme, and Dr. Mitchell’s role in it, this article provides an overview of the main themes in the most important of Dr. Mitchell’s historical public accounts. In the conclusion the article reflects on the wider insights that Dr. Mitchell’s accounts provide into how and why health professionals can be “sucked into the maw” of aggression (Rubenstein, 2020). The psychiatrist Lifton (2017) has noted that “we still lack a comprehensive accounting of medical and psychological behaviour during the Iraq War era. . .that would tell us a great deal more about vulnerabilities. . .among professionals in general”. This article attempts to use historical accounts to address some of this research gap.

## The psychologist^1^

The Report outlines the history of Dr. Mitchell’s involvement in the Enhanced Interrogation programme. Soon after 9/11 a perceived high-value detainee was captured by the U.S. security services. Dr. Mitchell, who was known to counter-terrorism officials from previous work, was brought in to “provide real-time recommendations to overcome. . .resistance to interrogation” (SSIR, 2014: 26). CIA headquarters flew out an interrogation team to question the detainee and Dr. Mitchell accompanied this team. His “initial role was to consult on the psychological aspects of the interrogation” (SSIR, 2014: 26). It was noted that Dr. Mitchell had expertise that was “so unique that we would have been derelict had we *not* sought [him] out when it became clear that CIA would be heading into the uncharted territory of the program” (SSIR, 2014: 32).

After the team’s assessment, meetings were held to discuss the possible use of novel interrogation methods. Based on his prior experience, Dr. Mitchell helped to create proposals for “using techniques derived from the U.S. military’s SERE school” (SSIR, 2014: 32), including sleep deprivation, waterboarding and constrained confinement. The idea was the techniques would facilitate the development of a rationalized (Deshotels et al., 2012) programme: one that was efficient and effective, safe, predictable and facilitated control. A senior CIA interrogator later said that the methods that were being proposed were similar to those used in “North Vietnamese physical torture” (SSIR, 2014: 33). The experienced interrogator Mark Fallon noted that waterboarding was originally used by Communist forces during the Korean War for propaganda purposes (Taub, 2019). Dr. Mitchell noted that “the safety of any technique lies primarily in how it is applied and monitored” (SSIR, 2014: 36).

Internal deliberations by CIA decision makers followed, after which the techniques that Dr. Mitchell helped to draft (which became known as Enhanced Interrogation Techniques) were approved for use. Dr. Mitchell was instructed to carry them out under supervision, including supervision by medical officers. The initial employment of Enhanced Interrogation was considered to be a success (it was noted that the purpose of the interrogations was to have “have broken any will or ability of subject to resist” (SSIR, 2014: 46) and provided a template for the future interrogation of high value detainees. It was recommended that psychologists should help to shape compliance of future detainees before detainees were questioned by debriefers (SSIR, 2014: 46).

The programme expanded after this initial Enhanced Interrogation event, eventually incorporating 119 detainees.^2^ An internal assessment of Dr. Mitchell’s performance (identified from thetorturedatabase.org) rated him as “exceptional” for quality of service, cost management, timeliness, and contract management/communication. It was noted that “Dr. Mitchell consistently met the highest standards of professionalism and competence”. However The Report suggested that Dr. Mitchell’s role, and that of other interrogators within the programme, was viewed more doubtfully by some. Some CIA medical officers for example were concerned about potential conflicts of interest, which were “nowhere more graphic than in the setting in which the same individuals applied an Enhanced Interrogation technique which only they were approved to employ, judged both its effectiveness and implicitly proposed continued use of the technique” (SSIR, 2014: 66).

In 2005 a private company was formed to provide operational psychologists, debriefers and security personnel at CIA detention sites. The Report argues that the CIA “outsourced virtually all aspects of the program” after this (SSIR, 2014: 11). The company was paid 81 million dollars for its services (Rosenberg, 2020a).

Revelations about Enhanced Interrogation, and the role of mental health professionals in it, began to come to light in the mid-2000s, and eventually the programme was brought to a halt in the late 2000s. The subsequent decade saw more disclosures about the programme’s activities, culminating in the release of The Report in 2014. The Report argued that the programme was “brutal and far worse than. . .represented to policymakers and others” (SSIR, 2014: 3). The Report noted, for example, that multiple detainees who were subjected to Enhanced Interrogation experienced “hallucinations, paranoia, insomnia, and attempts at self-harm and self-mutilation” (SSIR, 2014: 4). One detainee subjected to Enhanced Interrogation later wrote that “I reached a level of psychological, nervous and physical exhaustion that. . .I could officially have been declared psychotic” (Zubaydah, 2022). Others were “plagued by profound distress” for years after being interrogated (Risen, 2016; see also Apuzzo et al., 2016). Many individuals and groups, including those at the highest political levels, considered the Enhanced Interrogation programme to have employed serious and problematic levels of violence against the detainees in its custody (Gerstein, 2014; Hajjar, 2009; Physicians for Human Rights, 2021; SSIR, 2014; Tayler and Epstein, 2022; Welch, 2017). Physicians for Human Rights (2015) said that the psychologists helped to develop a programme “based on brutality and junk science” and “whose actions rank among the worse medical crimes in U.S. history”.

For his part, Dr. Mitchell, alongside other interrogators, consistently denied that Enhanced Interrogation constituted torture, and has also noted that the Enhanced Interrogation activities that he was involved in were authorized and approved at the highest levels. There was truth to this position. In the early years of the War on Terror the U.S. administration attempted to legally redefine what torture was, arguing that for an act to be considered torture it needed to result in pain equivalent to organ failure or death. The Convention Against Torture argues that for an act to be considered torture severe pain or suffering needs to be inflicted on a person. When talking about torture or Enhanced Interrogation, therefore, it is always important to consider what definition of torture or Enhanced Interrogation that an individual is operating under. The actions of an individual may well be viewed as one thing when considered under one definition, but not when considered under another.

## Accounts

As noted, Dr. Mitchell gave a number of public accounts about his role in, and perspectives on, Enhanced Interrogation. These accounts were provided over a number of years, and provided insights that were often lengthy, detailed and important. Dr. Mitchell gave conference presentations; a number of media interviews to both specialist online outlets and to national news broadcasters; interviews with national and international newspapers; and he also wrote a detailed memoir about his time in the Enhanced Interrogation programme. He said himself that his accounts were necessary because the authors of The Report “refused to interview any of the. . .contractors who actually have been involved in the program, including me” (Mitchell, 2016a). He said that he provided his accounts so that others would not “distort history” (Mitchell, 2016a). See Table 1 for a summary of these accounts. For this article, these accounts were thematically analysed following Braun and Clarke’s (2006) recommended approach.

**Table 1.** Dr. James Mitchell’s key accounts.

Account	Date	Details
Enhanced Interrogation: A Memoir	2016	Memoir. Most important account
Interview with Mark Steyn.	2017	1 hour long interview
Deposition	2017	Substantial interview (5 hours)
Interview with Mike Ritland.	2018	3 hours long interview
Interview with Vice News.	2014	25 minute interview
Interview at American Enterprise Institute	2016	1 hour long interview
Interview with Sean Hannity	2016	6 minute interview
Interview with Megyn Kelly	2014	12 minute interview
Interview with Sharyl Atkinsson	2014	5 minute interview
AFIO presentation	2017	40 minute presentation

Dr. Mitchell’s accounts were accepted within at least some social, security and political circles, and seen as the perspectives of an individual whose actions were unfairly demonized and had to make tough decisions, and sacrifice his own personal and professional well-being to protect and defend civilians from apocalyptic attacks. However his accounts were also criticized, including by mental health professionals. The psychiatrist Robert Lifton, for example, was shown an interview that Dr. Mitchell conducted with a major news network. Dr. Lifton’s (2015) response was:

That’s a shocking clip because it shows him slightly reluctantly admitting doing all those things. . .but the fact they’ll come on a network programme and describe it as something legitimate is another level of scandal.

A Psychologist who reviewed Dr. Mitchell’s memoir said “Mitchell’s dubious claims about the CIA’s abusive . . . ‘Enhanced Interrogation techniques’ are reasons enough to doubt his credibility” (Eidelson, 2017). The Psychologist also noted that “the choice he [Dr. Mitchell] made had calamitous effect. . .not only for the detainees who were tortured, but for the profession and the country as well” (Eidelson, 2017). Even an academic review that was more positively disposed to parts of Dr. Mitchell’s memoir (“an informative and interesting read”) argued that it had a tendency to “whitewash . . . . [It] especially suffers from his occasionally wrong statements which combine to undermine his generally valid argument” (Jens, 2017). Dr. Mitchell’s historical accounts and perspectives, like all accounts, therefore need to be considered carefully and situated in relation to wider historical research on this topic.

## Professional background

Dr. Mitchell was a successful psychologist before becoming involved in Enhanced Interrogation. He came from a modest background in Florida and joined the Air Force in the early 1970s seeking adventure (Shane, 2009). Stationed to Alaska, he became a bomb disposal expert. He noted that he became interested in how terrorists think while working in the bomb squad (Mitchell, 2017d). He consequently earned degrees in psychology (Shane, 2009). His CV (obtained from thetorturedatabase.org) indicates that he received his PhD in Clinical Psychology in 1986, after which he did significant additional professional training, including training in aeromedical psychology (Mitchell, 2014a). He published several academic articles, and presented widely on topics that were of interest to him. From the mid-1980s to the early 2000s he worked as a clinical psychologist, was a SERE (Survival, Evasion, Resistance and Escape) psychologist (where he was known as “Doc Mitchell” (Shane, 2009)) and was, in the late 1980s, Chief of an Adult Outpatient Psychology service. He developed interventions for HIV positive patients, worked with suicidal people, and conducted neuropsychological evaluations. He supervised psychology interns and psychiatry residents. As part of his SERE role he delivered and supervised resistance to interrogation training. He also worked in the area of hostage negotiation (Mitchell, 2016a, 2017a). He noted that he admired Arabic culture (Mitchell, 2016a).

In his work as a Psychologist Dr. Mitchell worked on a number of cases where he had to assess very violent individuals. For instance he noted one case where he assessed a man who had “stalked his wife. He had kidnapped her, duct taped her, beat her, raped her and then cut her eyelids off” (Mitchell, 2017a). In another case he noted that he assessed a man “who had sexually assaulted an eight year old girl with spina bifida” (Mitchell, 2017a). One of the lessons that Dr. Mitchell drew from these incidents is that the client of the psychologist, the one to whom the psychologist owes his or her loyalty and duty of care, is not necessarily the person or patient whom the psychologist is directly assessing or interacting with; “in some places the client is the person who comes to you, and in other cases your client is the Government. . .and you have to be clear, you know, who’s the client” (Mitchell, 2017a).

In interviews Dr. Mitchell generally presented as charismatic and knowledgeable, and someone with a strong sense of conviction. His silver hair and beard, his calming voice and accent, were noted, as was his professional confidence (though at the same time his aura of “visceral toughness” was also identified) (Shane, 2009). Dr. Mitchell appeared to be aware of his interpersonal aura and said that he employed it during interrogations: “I look like somebody’s uncle and I would go in and be the good guy” (Mitchell, 2018). In another interrogation he told a detainee “I’m an old man. It’s disrespectful to lie to me” (Mitchell, 2016a).

Dr. Mitchell and his wife were (as of 2018) married for close to 50 years (Mitchell, 2018). His wife’s father fought in Iwo Jima during World War 2 (Mitchell, 2016a). Dr. Mitchell was a supporter of Amnesty International, particularly their efforts to combat child abuse (Leopold, 2014). He was also a successful businessman with a strong entrepreneurial streak, developing a company that employed large numbers of people (Shane, 2009; Mitchell, 2016a). His non-professional activities tended towards what sociologists refer to as edgework activities, such as rock climbing, hiking, and high-intensity training (Mitchell, 2018). He noted that he liked to paddle out on the Myakka river in Florida and drift amongst the alligators (Mitchell, 2014a). Dr. Mitchell noted that he was “heavily armed, I’ve got a conceal-carry permit” (Mitchell, 2016b). He said that he was someone who could “protect myself”, and he was someone who could operate in a world of “trained killers and intelligence officers” (Mitchell, 2016a).

In 2001 he became an independent contractor and advisor for the CIA on interrogation and psychological profiling of detainees. He said that “I’m an ordinary guy who was caught up in extraordinary circumstances” (Mitchell, 2018).

## The personal context of Enhanced Interrogation

The violence of 9/11 deeply impacted Dr. Mitchell. He noted that:

I felt a tremendous sadness for the loss of life. I watched people jump to their death rather than burn alive. I heard comments about the number of people falling out of the sky. I watched as the building collapsed and people fled the dust cloud, covered with ash. . .I sat for a while on the floor in my living room, staring blankly at the TV as images of death and destruction and chaos flitted across the screen. I vacillated between profound sadness for the suffering of the victims of the attacks and a blood fever that made me want to get up right then, find the cowards who had ordered this, and fix it so that they could never do it again. (Mitchell, 2016a)

In several interviews Dr. Mitchell became emotional, to the point where it seemed he choked up or almost began to cry, when he reflected on the victims of 9/11. Years later, he noted that he became involved in Enhanced Interrogation “for the victims and the families”, because “I want[ed] to be part of the solution” (Mitchell, 2016a). He felt that in the moment of 9/11 “everything had changed, right for me it just completely changed my life” (Mitchell, 2014a). He thought of the passengers of Flight 93 “who bravely sacrificed their lives. . .I thought, if they can sacrifice their lives, I can do this” (Mitchell, 2016a).

Prior to 9/11 Dr. Mitchell had also been personally impacted by the actions of radicalized individuals. He said that one of his close friends, “one of the most gentle men I knew. . .very gentle”, had been murdered by Kashmiri separatists in the 1990s, which triggered in Dr. Mitchell an interest in apocalyptic violence and fundamentalism (Mitchell, 2017d). In a strange twist of fate, the group who committed that murder was also closely linked to a group that kidnapped the journalist Daniel Pearl; that latter group turned Daniel Pearl over to be murdered by one of the detainees who was eventually interrogated by Dr. Mitchell (2014a). In one interview Dr. Mitchell reflected on the detainee’s revelation concerning the murder of Daniel Pearl: “I can’t remember what he said about his hands. . .it’s something like ‘I cut his throat with these glorious hands’. . .absolutely no remorse at all” (Mitchell, 2018).

In many ways it appeared that Dr. Mitchell experienced his post 9/11 life as an attempt to understand, confront, and debride horror from the world; “Dr. Jessen and I were in the business of helping hunt down monsters” (Mitchell, 2018). The horrors of 9/11 stood out to him years after the event; “it was horrific that people had to choose between burning to death or jumping off buildings” (Mitchell, 2014a). Dr. Mitchell experienced dread at the thought of nuclear weapons being employed by terrorists (Mitchell, 2014b, 2016c, 2017a, 2018). He experienced at least some of his interactions with programme detainees as a confrontation with evil and death (Mitchell, 2016b), “the stuff of nightmares” (Mitchell, 2016a). Dr. Mitchell noted about one detainee that “he was very charming, immensely charming, but that is often how evil looks, right? If evil looks too evil, you can push back against it. If it’s charming, then you bring people into the fold, right?” (Mitchell, 2016b). He felt that his involvement in Enhanced Interrogation was necessary to prevent the creation of a dark, totalitarian world where:

It is normal and even desirable to burn. . .raze . . . destroy . . . slaughter people who aren’t like them while little children watch, or even take part in, the beheading, stoning, shooting, and burning of helpless victims. (Mitchell, 2016a).

## The institutional context of Enhanced Interrogation

Dr. Mitchell did not initially assume, when he became a CIA Contractor, that he was going to consult on detainee interrogations. It was not something that he said that he had sought out: “I didn’t knock on the gate and say, ‘let me torture people’” (Risen and Apuzzo, 2014; see also Mitchell, 2014a). Right from the start, though, he found himself working in the role of a psychological consultant on interrogations. He was told that “the gloves are off” and that there might be a “chemical, biological or nuclear” attack (Mitchell, 2016a). Dispatched with a team to a Black Site, he advised on the psychological aspects of a detainee interrogation. Dr. Mitchell and his colleagues were told to “think outside the box”, to “do everything and anything that was legal, to take it right up to the line” (Mitchell, 2016a). Dr. Mitchell said that he was very concerned that things were done legally (Mitchell, 2017a; see also Mitchell, 2017b). At first his job was to observe and assess the detainee’s resistance attempts. Very quickly Dr. Mitchell and other site staff, however, reported experiencing “intense pressure for results. There was a tremendous pressure not to let other Americans die” (Risen and Apuzzo, 2014; see also Mitchell, 2018). Dr. Mitchell reported that he and Dr. Jessen were asked by officials to assist in coming up with a proposal for harsher interrogation tactics. Dr. Mitchell indicated that he was initially reluctant to do this (Mitchell, 2017d). The two doctors though eventually “sat down at a typewriter together and we wrote out a list”, drawing from a list of tactics that they had worked with as SERE psychologists (Fink and Risen, 2017). Dr. Mitchell and Dr. Jessen felt that these tactics would be medically and psychologically safer to use than other potential tactics that non-psychologists might employ (Fink and Risen, 2017); “I said that if you are going to use coercive techniques, then don’t let people just freelance” (Risen and Apuzzo, 2014), “use. . .the things that have been used for 50 years with very few injuries” (Mitchell, 2018). He felt that Enhanced Interrogation techniques were unpleasant but prevented detainees from being subjected to worse techniques “made up on the fly” (Mitchell, 2016a). He recommended against the use of some Enhanced Interrogation techniques that he considered to be too dangerous (Mitchell, 2016a). The purpose of Enhanced Interrogation was to induce dread^3^ and “plant [in the detainee’s mind] the idea that there was a clear way to avoid it” (Mitchell, 2016a). Dr. Mitchell had an ambient sense that the organization was heading towards the use of aggressive interrogation tactics: “it was clear to me from walking the halls. . .it was clear that was the direction they were going” (Risen and Apuzzo, 2014; see also Mitchell, 2016a). When Dr. Mitchell proposed the use of Enhanced Interrogation techniques he said that he did not anticipate that he would be the person implementing them (Mitchell, 2017a).

Despite Dr. Mitchell’s pre-9/11 professional success and status, in many ways he experienced at least some of his role as an interrogator as being almost like a cog in a bureaucracy. Dr. Mitchell noted that “I’m just a guy who got asked to do something for his country by the people at the highest levels of government” (Leopold, 2014). As a contractor, the primary driver of his actions was the institution that he worked for. For instance, he rejected the idea that he was the architect of the Enhanced Interrogation programme saying “I don’t really think I was the architect of anything. . .we provided them with a list of techniques that they should consider, they eventually asked us if we would do them” (Mitchell, 2017a). Dr. Mitchell was careful and precise with his words. During an interview, when asked “you proposed that techniques from the SERE school be used?”, he responded: “I recommended that they consider using them” (Mitchell, 2017a). Another example: prior to an interrogation session, Dr. Mitchell consulted a lawyer about how to carefully verbally threaten a detainee; he was advised to make the threat conditional. So before telling the detainee “I will cut your son’s throat” he said “if another American child was killed” (Rosenberg, 2020b). Dr. Mitchell noted that eight children died on 9/11 (Rosenberg, 2020b).

In the Black Sites Dr. Mitchell noted that “I had zero decision-making power. My activities were controlled” (Mitchell, 2016a) – to the extent that he could be forbidden from using certain words in communication cables (Mitchell, 2016a). His lack of control could be detected in interrogation sessions. After waterboarding one detainee Dr. Mitchell felt that the detainee had become cooperative. However Dr. Mitchell then received instructions to continue to use Enhanced Interrogation. Dr. Mitchell reported that he did not like this, and questioned the necessity of this instruction; “the most senior CIA person in the country. . .we asked him if he could intercede with headquarters to get them to discontinue that use of-particularly of waterboarding- but of Enhanced Interrogations” (Mitchell, 2017a). He again received orders to continue to use the techniques. He was told “you guys have lost your spine. . . [the] blood of dead civilians are going to be on your hands” (Fink and Risen, 2017); that it will be “your fault. . .he’s turning you; you are not turning him” (Mitchell, 2016a); he said “I was told. . .that I’d become weak, that I must be starting to like these terrorists” (Mitchell, 2014b). Subsequent to being exposed to this enormous moral pressure, Dr. Mitchell decided that he had to waterboard the detainee for “one last time. . .if we didn’t, we probably would be replaced by other people who would not be as reluctant to use coercion as we were” (Mitchell, 2016a). Dr. Mitchell noted that “I felt sorry for him [the detainee]. I thought it was unnecessary. He had agreed to work for us” (Rosenberg, 2020c). Paradoxically, therefore, it seems that it was thought necessary to use Enhanced Interrogation techniques to prevent the use of Enhanced Interrogation techniques.

## The morality of Enhanced Interrogation

Dr. Mitchell’s accounts indicate that he did not find Enhanced Interrogation to be morally easy. He felt that it was a practice linked to “the dark side” and that “I knew I would be required to do things – harsh things-that some would view as unethical, maybe even monstrous” (Mitchell, 2016a). When he was initially asked to undertake Enhanced Interrogation techniques he said that he returned to his hotel room and had trouble sleeping. He found the stillness of the hotel room to be difficult to deal with (Mitchell, 2016a). He was conscious that many people would refuse to support Enhanced Interrogation and he noted that “I respect that” (Mitchell, 2016a). His thoughts, though, returned again and again to 9/11, and the risk of the use of nuclear weapons by terrorists. Two questions circled in his mind: Could I do it? And should I do it? (Mitchell, 2016a).

Ultimately, he came to the decision that it was ethically acceptable, though not ethically easy, to engage in Enhanced Interrogation. There appeared to be several dimensions to his ethical reasoning. The first of these was a type of means-end calculation. He felt that morally it was necessary to protect thousands from the actions of a murderous few (Rosenberg, 2020a); “I thought about it long and hard” (Mitchell, 2018), it was “a patriotic duty” (Mitchell, 2017a). Secondly he felt that Enhanced Interrogation methods were “lawful, authorized and carefully monitored”, which made them more acceptable to him (Mitchell, 2016a) (the relationship between morality and legality can be a complicated question). Thirdly, Dr. Mitchell also felt that he had the scientific skills and training to do Enhanced Interrogation in a safe and effective manner, and felt that it would have been unethical to leave Enhanced Interrogation to less skilled individuals as the risk of detainees being harmed would increase (Mitchell, 2016a). He expressed concerns about detainee suffering; “it bothered me a lot. You never like to watch a human being suffer. . .you wouldn’t want anyone doing waterboarding that liked to watch human beings suffer” (Mitchell, 2014c). He also noted that “I didn’t like it [waterboarding]. I didn’t enjoy it. I wasn’t thrilled by it” (Mitchell, 2014b). He felt that Enhanced Interrogation sometimes put him in a situation where “I had to choose the least bad amongst several bad choices” (Mitchell, 2016a).

Dr. Mitchell said that he continued to reflect on the morality of Enhanced Interrogation after the programme began.

Bruce and I then went for a long walk. This would become our habit after sessions using EITs (Enhanced Interrogation Techniques), especially the ones involving waterboarding. We didn’t like using EITs, and we used the walks to think about what we were being asked to do and consider whether we were making the right decision in continuing. I often felt that I was balancing my sense of morality against the cost of innocent lives. Because there was credible intelligence that another wave of catastrophic attacks was imminent, I couldn’t bring myself to soothe my conscience by putting the lives of others at risk. Neither, I think, could Bruce. But we had a line, and we checked every day to be sure we hadn’t crossed it. (Mitchell, 2016a).

As noted above, however, at certain points when Dr. Mitchell reached what he considered to be a moral line in the sand he could experience significant pressure to cross it.

## Science

Dr. Mitchell’s accounts made frequent reference to science, especially psychological science. An important part of his identity was his role as a clinical psychologist (Mitchell, 2017a); “as a psychologist with a Ph.D. in clinical psychology. . .I knew how to establish rapport and ask questions” (Mitchell, 2016a). One of the companies that he founded was called “Knowledge Works” (Mitchell, 2017a); another was called “Mind Science” (Mitchell, 2017a). At the SERE School he noted that he worked in the “resistance training laboratory” to prevent SERE trainers from experiencing “abusive drift” and, potentially, torturing students (Mitchell, 2017a). He noted that attempts by the CIA to create more interrogators like himself and Dr. Jessen failed because what “they didn’t realize is that both of us have Ph.D.s in psychology” (Mitchell, 2018). The conceptual language that he used to interpret detainees’ behaviours drew on concepts such as splitting, bridging, conditioning and anchoring (Mitchell, 2016a, 2016b); he felt that some non-psychological interrogators made mistakes because they employed Enhanced Interrogation techniques without fully understanding the psychological concepts that underpinned them (Mitchell, 2016a). This increased the risks of unintentionally producing profound despair in detainees (Mitchell, 2017a). He found that when done successfully many interrogations could settle into a routine that was “for the most part like doing therapy” (Mitchell, 2018). He also noted that “it was pretty interesting to watch” how some detainees adapted to Enhanced Interrogation techniques (Mitchell, 2014c).

However his scientific identity was also attacked. A psychiatrist said of Dr. Mitchell and his colleague Dr. Jessen: “my impression is that they misread the theory. . .they’re not really scientists” (Carey, 2014). Dr. Mitchell said that he was aware when he decided to become involved in Enhanced Interrogation that “if I did these interrogations it’s [mental health work] gone. . .I knew within my profession I would be ostracized” (Mitchell, 2018). He felt that “the bulk of psychologists would probably object, you know. So what I thought was it’s highly probable that I’m not going to go back to, you know, doing mental health work” (Mitchell, 2017a). Eventually Dr. Mitchell resigned from his professional association partly because “I didn’t like the stance” that they took on the role of psychologists in custodial interrogations (Mitchell, 2017a).^4^ He noted “those people are not part of my life. I don’t care what they think” (Mitchell, 2016b). He also said that he had to be careful when talking about scientific experimentation in particular “because the ACLU sues me, one of the things they sued me about was that I treated these guys like dogs” (Mitchell, 2018).

Dr. Mitchell could himself be bemused by some of the psychological approaches used by other interrogators in the programme. One other interrogator for instance was described as being “unnaturally obsessed with. . .neuro-linguistic programming” which Dr. Mitchell said might as well have been “magic. . .or a Jedi mind trick” (Mitchell, 2016a). Another psychologist was noted by Dr. Mitchell to have lost his objectivity during a detainee assessment:

During the visit he [the psychologist] started telling X [the detainee] he would burn in hell for killing innocent people. He then launched into a. . .harangue, cursing X, . . . screaming at him. X started screaming back. The guards had to pull the psychologist out. . .I did see [the psychologist] at other Black Sites evaluating other detainees. If it had been up to me, he would not have had any contact with detainees. (Mitchell, 2016a)

## Controlled violence

Enhanced Interrogation, if done correctly, was viewed as a very controlled, calibrated form of violence. Dr. Mitchell felt that it was not torture; “If it was torture, I would be in jail” (Mitchell, 2014b). While he felt that Enhanced Interrogation was uncomfortable and potentially risky it was, if done correctly, ultimately safe and effective (Mitchell, 2018); “I think you can do it in a way that it constitutes torture, I think you do it in a way that it constitutes training. . .it’s like every tool” (Mitchell, 2014a). Dr. Mitchell noted that “the waterboard induces fear and panic. It is scary and uncomfortable but not painful” (Mitchell, 2016a). Dr. Mitchell also noted, though, that “to be candid with you, if you’re going to break somebody’s legs or waterboard them, they probably would prefer you break their legs” (Mitchell, 2014a) – though in yet another interview he said that this was hyperbole (Mitchell, 2017a). Dr. Mitchell said that he adjusted the length of waterboarding sessions to increase safety (Mitchell, 2016a).

Overall, Enhanced Interrogation sessions, when done well, were like “well-oiled machines”^5^ (Mitchell, 2018) with medical personnel and additional Psychologists present to ensure a detainee’s well-being. The “safeguards were built in” (Mitchell, 2017a); “everyone knew that anyone could stop an interrogation at any time for safety reasons” (Mitchell, 2016a). The interrogation team practiced how to respond to medical emergencies. . .practiced, practiced, practiced” (Mitchell, 2016a). A fully stocked emergency room crash cart was stationed outside of one interrogation session (Mitchell, 2016a). Dr. Mitchell felt that if the techniques were implemented correctly they would not create long-term physical or psychological harm (Mitchell, 2017a). Furthermore he felt that the programme was highly standardized. For example when loud music was played in order to psychologically disrupt detainees, the music was governed by noise exposure standards (Mitchell, 2016a) (the programme played loops of a little girl screaming “Daddy! Daddy! The bad man is hurting mommy!” (Mitchell, 2016a)). Within interrogation sessions considerable attention could be paid to technical standards such as the angle of waterboard gurneys (to make sure that water did not go down a detainee’s lungs) (Mitchell, 2016a, 2018). At the same time, he noted that he could be taken by surprise after some of the unanticipated effects of Enhanced Interrogation, such as detainees vomiting after waterboarding (Mitchell, 2016a).

## Uncontrolled violence

However Dr. Mitchell’s accounts also contain details of uncontrolled, apparently unauthorized violence being used by other interrogators. At a certain point The Report notes that an additional interrogation group in the CIA became involved with conducting Enhanced Interrogations; a lawyer who was directly involved in the program claimed that she “was never sure what group. . .was responsible for interrogation activities” (SSIR, 2014: 64). Dr. Mitchell felt that interrogators within the parallel group used techniques that violated guidelines and safety protocols; he said that some of the techniques that they used “distressed and concerned me” (Mitchell, 2016a).

In one instance he noted that an interrogator used a stiff brush on a detainee to scrub him “alternating between his ass and balls and then his mouth and face” (Mitchell, 2016a). In another instance an interrogator threatened a detainee with a “handgun and a drill” (Mitchell, 2016a). Dr. Mitchell shared his concerns after one such interrogation and “I was told that. . .I had no say. . . at what happened at this Black Site or anywhere else in the world” (Mitchell, 2016a). Dr. Mitchell felt that his safety concerns were disregarded because of his status as an independent Contractor: “I can’t tell you how many times I’ve been told. . .what you think” is not important (Mitchell, 2018); “we were contractors. . .with no authority” (Mitchell, 2016a). In one instance when Dr. Mitchell asked to speak to Headquarters for legal advice he was told that was “not going to happen” (Mitchell, 2016a), that he was not going to call anyone “especially the fucking lawyers” (Mitchell, 2016a) and he said that it was implied to him that if he made trouble he would be framed as a “vindictive troublemaker, something I discovered later that they did” (Mitchell, 2016a). He said that he himself was effectively almost taken “prisoner at a Black Site” so that he would not be able to report his concerns (Mitchell, 2016a). Once he left the Black Site Dr. Mitchell and Dr. Jessen discussed if “we ought to cut our ties with the programme” (Mitchell, 2016a). However he felt that he could have a positive influence on the programme, so agreed to return to it. During a prisoner transfer Dr. Mitchell became concerned about the way two detainees were being handled and was told to “mind my own fucking business” (Mitchell, 2016a).

Dr. Mitchell’s accounts also suggest that medical supervision of interrogations could be suboptimal. At one point in the programme Dr. Mitchell noted that a detainee’s wrists became infected. When he told the medic, the medic responded “I’m not here to provide medical care for fucking terrorists” (Mitchell, 2016a). In one account Dr. Mitchell himself attempted to unsuccessfully stop an interrogation for medical reasons:

“The things the interrogators are doing have not been approved by the Justice Department, and they should stop,” I whispered to the guard. . . “I think they are going to dislocate X’s shoulders. Headquarters policy is to stop interrogations when someone raises a concern about safety”. . . The guard nodded, walked to where the chief interrogator was standing glaring at me, and whispered something in his ear. . .the chief interrogator pointed to the door, hissed for me to get out, and instructed the guard to escort me completely out of the interrogation room. The interrogation was being observed by medical personnel and several others on closed circuit TV. I was surprised medical personnel had not intervened and said so. I expressed my concerns to them and the guards. I got a “so what can you do?” look from the medic. The guards said they were worried, but their hands were tied because they had been told the chief interrogator called all the shots (Mitchell, 2016a).

For his efforts, Dr. Mitchell reported that he was told that “it was a mistake to involve me because I was a psychologist and a bleeding-heart liberal who cares. . .about the feelings of a fucking terrorist” (Mitchell, 2016a). Dr. Mitchell was clear that from his perspective he attempted to report abuses whenever he saw them,^6^ to clean out “rogue elements” (Mitchell, 2016a). He also felt that, as time went on, the programme’s adherence to its own guidelines improved, and that deviance within the programme was increasingly corrected when it appeared (Mitchell, 2016a).

The Report also noted that some detainees were forcibly subjected to practices such as rectal feeding, which has a problematic history within mental health (Sammet, 2006). While this practice was not part of Enhanced Interrogation and people like Dr. Mitchell were not associated with it, it did form part of the wider violent context in which detainees were embedded.

## What exactly was the Enhanced Interrogation programme?

Dr. Mitchell’s accounts raised an interesting question: what exactly was the Enhanced Interrogation programme? Dr. Mitchell argued that the safety concerns raised about the programme in The Report could, in some cases, be considered separately as they were not, from his perspective, part of Enhanced Interrogation. He argued for instance that that detainee who died was “not a detainee in the high-value programme that I was involved in, and the CIA officer responsible for questioning him was not an interrogator from that programe” (Mitchell, 2016a). He further noted that:

Allegations about detainees being forced to stand on broken bones and chained for weeks in total darkness in what amounted to a freezing dungeon were made. If such things happened (and I cannot confirm that they did), most of the problems did not involve program interrogators and happened in places that were not under the program’s operational control.

However as he himself noted, Enhanced Interrogation activities appeared to involve multiple competing units and extended beyond his particular group and immediate context. Different units and personnel could have “very different ideas about how Enhanced Interrogation Techniques should be used to acquire actionable intelligence” (Mitchell, 2016a). How someone defines and sees what the programme is will have implications for their ethical decision-making and ultimately their sense of responsibility for the programme’s actions. It was fair to say that the programme’s critics were probably concerned and horrified about the whole thing, the entire programme, irrespective of whether it was the part of the programme that was following the guidelines closely or the part that was, from Dr. Mitchell’s perspective, freelancing.

## Consequences and legacy

For a number of years post-9/11 Dr. Mitchell occupied an important role in one of the most important intelligence programmes in one of the most important institutions in the world. However his “fall from official grace [was] as swift as [his] rise” (Shane, 2009). The Enhanced Interrogation programme was brought to a stop in the second half of the 2000s, and information about Dr. Mitchell’s role was gradually revealed in the years afterwards. His involvement in the programme was condemned by some health professionals and groups. Dr. Mitchell reflected over the years on his involvement in the programme, and on some audience reactions to it. He noted while “I’m proud of the work we did. . .I do have some bitterness about it” [how the interrogators were treated]. (Mitchell, 2017d). He felt that he and Dr. Jessen especially were “thrown under the bus” (Mitchell, 2018; see also Leopold, 2014, Mitchell, 2017c). He felt that “we were being set up to be the fall guys” (Mitchell, 2016a). Dr. Mitchell directed particular anger towards The Report which he argued was “cherry-picked, taken out of context and framed to produce a misleading narrative” (Mitchell, 2016a). He felt that The Report was about “denigrating the program and destroying the reputations of the people involved” (Mitchell, 2016a). Dr. Mitchell noted “I’m poison to those people” (Mitchell, 2018), and a sympathetic interviewer told him “to a lot of people. . .you are Hannibal Lecter” (Mitchell, 2017d). When another interviewer informed him that “you have to be an incredibly tough person to survive [that type of response]” Dr. Mitchell appeared to become momentarily emotional at the supportive words (Mitchell, 2017d). Dr. Mitchell frequently recounted an interaction that he had with a detainee where he said that that the detainee warned him that at some point the world would turn on Dr. Mitchell and the other Enhanced Interrogators and the interrogators would be scapegoated for their involvement in the programme (Mitchell, 2016a).

Dr. Mitchell experienced serious safety consequences himself as a result of his identity becoming public. He noted “we’ve all these crazies running around and making threats” (Mitchell, 2014c). He said that he received credible death threats from ISIS, who asked for a jihadist volunteer to behead him (Mitchell, 2016a).

Dr. Mitchell’s accounts indicated that he was highly concerned about future attacks by terrorists. He felt that the West was at war with terrorists for whom killing was “an act of worship. . .when they crucify. . .it was an act of love” (Mitchell, 2017d). He noted that “I don’t think we are safer than before 9/11” (Mitchell, 2017c). However he felt that the West had become “risk-averse” (Mitchell, 2018) and was failing in its efforts to combat dangerous, viral ideologies (Mitchell, 2016b). In one account he noted that terrorists felt that “the real war will be fought in the minds of the people” (Mitchell, 2017c). He felt that there was no point “standing on our moral high ground looking down into our smoking hole that used to be several blocks in Los Angeles” (Mitchell, 2016b). He also felt that there should be a discussion of the “rules of engagement [imposed] on the lives of those who protect us. Rules of engagement that can endanger their lives or the missions success. So we have some hard decisions” (Mitchell, 2017c).

## Discussion

Dr. Mitchell consistently denied that the Enhanced Interrogation programme that he was involved in committed torture. In this he was supported by many senior officials who had oversight over the programme. He said that “I’m not for legally torturing anybody. If it’s torture, don’t do it. If it’s illegal, don’t do it” (Mitchell, 2016b). He noted in the years after the programme ended that “we were at war. Our actions were necessary, legal, authorized and helped save lives” (Mitchell, 2016a). Dr. Mitchell also noted, though, that The Report “is filled with instances where they think things got a little out of hand – well, a lot out of hand” (Mitchell, 2014c) and that “some things happened. . .that weren’t part of that programme. . .[that were] probably torture. But it wasn’t part of the official programme” (Mitchell, 2016b). It is fair to say that Dr. Mitchell was, at the least, highly concerned about some Enhanced Interrogations that he witnessed. Despite his criticisms of The Report, what he described sometimes witnessing in his historical accounts was as – or even more – concerning than the information revealed within The Report itself.

A number of reasons have been previously identified for why health professionals might become involved in aggressive interrogations. Dr. Mitchell, as noted, always took great care to differentiate Enhanced Interrogation from torture. However, even if the two practices are seen as separate, perhaps categorically different, perhaps existing along a spectrum, some of the same factors that drive health professional involvement in torture also seem relevant to the factors that drive health professional involvement in Enhanced interrogation. These include having specialized scientific knowledge that might be of use to the state in a time of emergency (Welch, 2009, 2017); a sense that involvement in aggressive interrogations might help to save the lives of civilians and detainees; a strong sense of the righteousness of the cause and of personal and group morality; and even being employed by the security services in a wartime context. Some people involved in Enhanced Interrogation were covered by a contract giving them multi-million dollar indemnification against criminal prosecution (SSIR, 2014: 169). The risk of this is that it potentially makes it easier for interrogators to cross lines and limits as the personal risk of doing so is substantially reduced.

Dr. Mitchell’s historical accounts also suggest another possible reason for why health professionals might become involved in Enhanced Interrogation related behaviours: what (Lifton, 2003) refers to as a “death imprint”. One of the most striking features of Dr. Mitchell’s accounts was his emotional response whenever he talked about the victims of 9/11. He often had to stop for a moment to collect himself in order to avoid becoming overwhelmed by emotion. Lifton (2003) argues that when people, including health professionals, encounter apocalyptic death and violence it can have important significance for them and for their later actions. People can experience death anxiety, fearing recurrence of the disaster and the individual’s inability to prevent it from happening again. They can also experience death guilt, which can haunt the survivor or witness, and involve a sense that the individual has a debt to the dead that can never be repaid. Intense death anxiety about apocalyptic risks, such as the use of nuclear weapons, can drive urgent risk orientated actions (such as Enhanced Interrogation activities), which themselves perhaps could be viewed as a way of demonstrating an attempt to control, confine and, perhaps, master death, to keep it contained and away from the world of the living. Such anxieties, if they are indeed present, could also be intensified from repeated interactions with apocalyptic individuals who “love death more than life” (Mitchell, 2017c) and whose goal is to destroy the world. As noted, he himself felt that his encounters with some detainees was “the stuff of nightmares” (Mitchell, 2016a). Death guilt could also be a motivator for these behaviours. However a problem with death guilt in this type of context is that the courage that some people show during apocalyptic events, and the horrors that some people experience, are almost limitless. Using their sacrifice and death as a guide for one’s own actions means that that there is potentially no limit on what the individual can or should do in response, as it is impossible to live up to the selflessness of, and pain experienced by, the dead.

The risk of unrecognized death anxiety and death guilt in a situation where Enhanced Interrogations are occurring would seem to me to be real. From a sociological perspective, Enhanced Interrogations could be interpreted as a type of edgework activity. An edgework activity is one where an individual, or group, is engaged in a dangerous course of action and needs to use significant skill and ability to do something that is risky, without losing control over risk and falling into chaos. Death anxiety and guilt would be problematic in edgework situations such as Enhanced Interrogation as they could lead people to engage in actions that are either too risky or too aggressive. While Dr. Mitchell did not describe himself becoming overwhelmed by emotion within Enhanced Interrogation sessions, he did observe some other interrogators appearing to become so; he noted that one interrogator “had let his feelings get in the way of his objectivity” (Mitchell, 2016a).

Dr. Mitchell’s accounts also indicate the problems experienced by health professionals who want to standardize and control Enhanced Interrogation type activities. Here Dr. Mitchell’s own accounts of his concerns were different from how he and other interrogators were often represented (e.g. as “Hannibal Lecter”, “monsters” etc.). Dr. Mitchell reported having strong safety concerns and that he was prepared to try to act on them. However despite his safety concerns, and despite his elite health professional background, in some instances both he and medical professionals within the programme appeared to be powerless to prevent what he considered to be violations from occurring – even when those violations were occurring directly in front of him. It was clear that, from his perspective, within the context that he was operating, obedience to authority was a key principle. If someone in a position of power decided to deviate from guidelines there appeared to be little that could immediately be done, apart from attempting to appeal to a higher level of authority – which could, as he found out, be prevented. Dr. Mitchell’s outsider/contractor status, far from acting as a break on the behaviour of participants actually seemed to mean that his safety concerns could be disregarded by some organizational insiders, at least in some instances. This undermines one of the key rationales for the presence of a psychologist or psychiatrist during Enhanced Interrogations, which was to prevent “abusive drift. . .the tendency for the intensity of physical coercion to escalate in the absence of careful supervision” (Mitchell, 2016a).

Dr. Mitchell’s accounts also provided hints that health professional presence within interrogations might have actually, unintentionally, enabled deviance within the programme to occur, to maintain – simply by their presence (their participation was not necessary) – what Lifton refers to as “the atrocity-producing situation”. For instance, one of the interrogators who Dr. Mitchell considered to be problematic told Dr. Mitchell that “you are not leaving. You have to be on-site in order for us to do interrogations. So you’re not going anywhere” (Mitchell, 2016a). Dr. Mitchell noted that “I thought briefly about refusing” but ultimately decided to “accept the role of psychologist” (Mitchell, 2016a). He noted that he felt that if he walked “that would not have stopped the interrogations. . .it would just mean that there would be no psychological monitoring” (Mitchell, 2016a). However by staying within the situation there is always a risk then that the behaviour that is of concern will become normalized and the situation seen as acceptable, especially if the health professional had initially objected to it. It is possible that if mental health professionals and psychologists within the programme were operating under a different starting assumption^7^ (“the programme has become deviant and needs to end now, irrespective of the consequences”), if they were less caught between what they felt were competing ethical imperatives (i.e. “this is wrong, but if I go now there is a risk that people might end up hurt or even die”), then they might have been able to end the deviance more effectively and quickly.

As noted earlier, while Dr. Mitchell’s historical accounts are useful and important for providing insider insights into the Enhanced Interrogation programme, they were also viewed skeptically by some. Some health professional groups at the time condemned Dr. Mitchell’s activities; “James Mitchell is a living example of the harm doctors and psychologists do when they deviate from. . .ethical obligation. . .it is time for every health professional and every citizen to speak out in response to James Mitchell’s justifications” (Physicians for Human Rights, 2020). A number of doctors noted that while the psychologists in the Enhanced Interrogation programme were concerned about trying to maintain standards in relation to certain aspects of the situation that they were in, they failed to really grasp that it was the entire situation that might have been unethical and problematic. Dr. Mitchell’s historical accounts, therefore, need to be considered carefully. It is important to read them alongside, rather than instead of, The Report, as well as alongside critiques of the programme produced by organizations such as Physicians for Human Rights.

There are a number of areas where important information is somewhat unclear or could be more present in Dr. Mitchell’s accounts. The first of these concerns how interrogators within the project actually assessed and analysed the effectiveness of what they were doing. For example Dr. Mitchell noted at one point that he estimated that it would take about 30 days to know whether or not Enhanced Interrogation would work on a detainee (Mitchell, 2016a), but it is uncertain where that number came from. He also noted “I’m not the best guy to decide whether or not they’re [Enhanced Interrogation Techniques] effective for producing actual intelligence. . .the people who are experts. . .I relied on them to tell me if from their perspective it was effective” (Mitchell, 2017a). Researchers independent of the Enhanced Interrogation programme have argued that the types of techniques that the programme used have historically been suboptimal for obtaining accurate intelligence (Duke and Van Puyvelde, 2017). A senior CIA interrogator noted that tactics similar to Enhanced Interrogation Techniques were only designed to “extract confession for propaganda purposes. . .[from those] who possessed little actionable intelligence” (SSIR, 2014: 33). It is an important consideration if Enhanced Interrogators were not evaluating the analysts who were evaluating their activities, and thereby taking their statements about effectiveness at face value.

Additionally, I think that more information would have been useful about how Dr. Mitchell and other interrogators assessed the quality of the information that they were helping to generate. Dr. Mitchell noted that analysts told him that the information that he helped to generate had helped to “disrupt future attacks and capture terrorists who were still at large. I believed them” (Mitchell, 2016a). However the Senate Report argued that some detainees fabricated information and others produced no intelligence. A framework describing how interrogators assessed quality would have been helpful for the historical record.

There is also a lack of evaluation within the accounts about what interrogators considered to be the actual real level of risk of nuclear violence by terrorist groups. Dr. Mitchell and other interrogators were told “that the next attack might involve chemical, biological or nuclear devices”. . .“that they had credible evidence that Al-Qaeda was planning another catastrophic attack and that could potentially involve a nuclear weapon” (Mitchell, 2017a). It is unclear though how interrogators assessed these statements when they were presented to them, or if they accepted them at face value. It is obviously not the case that there is no risk that terrorists and apocalyptic groups would seek out nuclear weapons (Bunn, 2021); and it is obvious that some people are willing to kill huge numbers of other people for their beliefs (Pluta and Zimmerman, 2006). However how realistic were these fears of terrorists getting their hands on nuclear weapons? How immediate were they? Dr. Mitchell noted that his background was as a “bomb disposal guy” (Mitchell, 2016b). His perspective on the nuclear device information that was presented to him, given the practicalities that would be involved in developing such a weapon, getting it secretly across an ocean, getting it to a city and so on, would have been useful.

Dr. Mitchell’s accounts also seem to suggest that the processes for resignation from the programme might have been unclear; this is important given the deviant behaviour that Dr. Mitchell felt emerged within it at several points. Part of the issue might have been the quickness with which Dr. Mitchell was recruited into the programme; he noted that “because of time constraints [my initial contract] was written in ballpoint ink on the front size of a single sheet of yellow legal paper” (Mitchell, 2016a). At points he noted that attempting to get out of a black site and report misconduct was like falling “down a rabbit hole” (Mitchell, 2016a). Once out, as noted earlier, he told Dr. Jessen that they ought to sever their connections to the programme (Mitchell, 2016a). However he was quickly drawn back into it when he was told that he could save lives. It is unclear the extent to which processes and procedures were in place to allow interrogators to cleanly exit the programme and make formal complaints; from these accounts, such processes, at least at certain points, might have been lacking or at least functionally amorphous.

From the perspective of his critics, Dr. Mitchell and his colleagues were almost something like false scientific prophets, professionals who promised salvation through an unreal catechism of aggression. His supporters, in turn, viewed him and other Enhanced Interrogators as a type of Cassandra, professionals who saw truly and clearly and were scapegoated for the clarity of their sight and their willingness to do what they felt had to be done to prevent future apocalyptic attacks. Dr. Mitchell for his part made reference to Star Wars in some of his accounts. Interestingly, while he has referred to detainees as “Jedis^8^” and “Yoda” (Mitchell, 2016b), he referred, as noted earlier, to the activities of the Enhanced Interrogation programme as being part of “the dark side” (Mitchell, 2016a). Mental health professionals who are thinking of becoming involved in Enhanced Interrogation type activities in future wars should reflect on Dr. Mitchell’s perspectives and experiences, which are of significant historical importance, and keep in mind something that Yoda once said: When you look at the dark side, careful you must be. For the dark side looks back.

## Notes

1. Unless otherwise noted, all direct quotations in this section are from *The Report*.

2. Dr. Mitchell appears to have worked with a smaller sub-section of this 119 figure.

3. Even the locations in which Enhanced Interrogation occurred, the Black Sites, have connotations of horror and dread (McClanahan and Linnemann, 2018).

4. The American Psychological Association wrote to the Texas State Board of Examiners of Psychologists on June 30th, 2010. They said that “the allegations regarding Dr. Mitchell’s conduct are unique in the scope of misperception and harm they have caused regarding the public’s understanding of the profession of psychology and its ethical principles. . .acts such as waterboarding. . .are explicit violations of APA policy. . .if any Psychologist member of APA were proven to have committed the alleged acts. . .it would be our expectation that the individual’s state license to practice Psychology would be revoked”.

5. At points Dr. Mitchell noted that detainees could begin to figure out and resist Enhanced Interrogation Techniques. Interrogators might change their strategy then. However it is unclear if he felt that detainees could continually figure out and adjust to the patterns and practices that were applied to them.

6. In his pre-Enhanced Interrogation work as a SERE Psychologist Dr. Mitchell “was known for enforcing the safety of interrogations. . .he eliminated a tactic called ‘manhandling’ after it produced a spate of neck injuries” (Shane, 2009).

7. Soldz (2018) uses the term “Telos” to describe the starting goal or assumption that each profession is and which recommends what it should do.

8. Dr. Mitchell also noted that when the Jedi façade was discarded by detainees “a crude thug would emerge” (Mitchell, 2016a).

## References

[bibr1-0957154X251358448] Apuzzo M Fink S Risen J (2016) How U.S. torture left a legacy of damaged minds. Nytimes. Available at: https://www.nytimes.com/2016/10/09/world/cia-torture-guantanamo-bay.html (accessed 18 February 2022).

[bibr2-0957154X251358448] Braun V Clarke V (2006) Using thematic analysis in psychology. Qualitative Research in Psychology 3(1): 77–101.

[bibr3-0957154X251358448] Bunn M (2021) Twenty years after 9/11, terrorists could still go nuclear. Bulletin of the Atomic Scientists https://thebulletin.org/2021/09/twenty-years-after-9-11-terrorists-could-still-go-nuclear (accessed 9 April 2026).

[bibr4-0957154X251358448] Carey B (2014) Architects of CIA interrogation drew on psychology to indice helplessness. Nytimes. Available at: http://www.nytimes.com/2014/12/11/health/architects-of-cia-interrogation-drew-on-psychology-to-induce-helplessness.html (accessed 4 February 2022).

[bibr5-0957154X251358448] Deshotels T Tinney M Forsyth C (2012) McSexy: Exotic dancing and institutional power. Deviant Behavior 33: 140–148.

[bibr6-0957154X251358448] Duke MC Van Puyvelde D (2017) What science can teach us about ‘enhanced interrogation’. International Journal of Intelligence and Counterintelligence 30: 310–339.

[bibr7-0957154X251358448] Eidelson R (2017) Heart of darkness: Observations on a torture notebook. Counterpunch. Psychology Today. Available at: https://www.psychologytoday.com/ie/blog/dangerous-ideas/201701/heart-darkness-observations-torture-notebook (accessed 13 February 2022).

[bibr8-0957154X251358448] Fink S Risen J (2017) Psychologists open a window on brutal C.I.A. interrogations. Nytimes. Available at: https://nyti.ms/2tMQ1l0 (accessed 24 February 2022).

[bibr9-0957154X251358448] Gerstein J (2014) Obama: ‘We tortured some folks. Available at: https://www.politico.com/story/2014/08/john-brennan-torture-cia-109654 (accessed 5 February 2022).

[bibr10-0957154X251358448] Hajjar L (2009) Does torture work? A sociolegal assessment of the practice in historical and global perspective. Annual Review of Law and Social Science 5: 311–345.

[bibr11-0957154X251358448] Jens E (2017) A review of enhanced interrogation. Studies in Intelligence 61(3): 173–175.

[bibr12-0957154X251358448] Johnson D Mora A Schmidt A (2016) The strategic costs of torture: How enhanced interrogation hurt America. Foreign Affairs 95(5): 121–132.

[bibr13-0957154X251358448] Leopold J (2014) I’m just a guy who got asked to do something for his country. The Guardian. Available at: https://www.theguardian.com/world/2014/apr/18/james-mitchell-cia-torture-interview (accessed 1 March 2022).

[bibr14-0957154X251358448] Lifton R (2003) Superpower Syndrome. Nation Books.

[bibr15-0957154X251358448] Lifton R (2015) Psychologists who aided torture should be charged. Democracy Now. Available at: https://www.youtube.com/watch?v=klAppx2gy_I&ab_channel=DemocracyNow%21 (accessed 14 February 2022).

[bibr16-0957154X251358448] Lifton R (2017) Malignant normality. Dissent Magazine. Available at: https://www.dissentmagazine.org/article/malignant-normality-doctors (accessed 17 February 2022).

[bibr17-0957154X251358448] McClanahan B Linnemann T (2018) Darkness of the edge of town: Visual criminology and the black sites of the rural. Deviant Behavior 39(4): 512–524.

[bibr18-0957154X251358448] Miles S (2020) The Torture Doctors. Georgetown University Press.

[bibr19-0957154X251358448] Mitchell J (2014a) The architect of the CIA’s Enhanced Interrogation programme, James Mitchell. Vice News. Available at: https://www.youtube.com/watch?v=MmNUi0itl-8&ab_channel=VICENews (accessed 15 January 2022).

[bibr20-0957154X251358448] Mitchell J (2014b) Man who Waterboarded 9/11 mastermind. The Megyn Kelly Show. Available at: https://www.foxnews.com/transcript/man-who-waterboarded-9-11-mastermind-if-it-was-torture-i-would-be-in-jail (accessed 12 January 2022).

[bibr21-0957154X251358448] Mitchell J (2014c) Sharyl Attkinson interviews the man who Waterboarded 9/11 matermind. The Daily Signal. Available at: https://www.youtube.com/watch?v=qUI3QoxE9fk&ab_channel=TheDailySignal (accessed 3 February 2022).

[bibr22-0957154X251358448] Mitchell J (2016a) Enhanced Interrogation. (New York: Crown Forum).

[bibr23-0957154X251358448] Mitchell J (2016b) Inside the Islamist Terrorist’s Mind: A Conversation with Former CIA Interrogator James Mitchell. American Enterprise Institute. Available at: https://www.aei.org/events/inside-the-islamist-terrorists-mind-a-conversation-with-former-cia-interrogator-james-mitchell/ (accessed 17 January 2022).

[bibr24-0957154X251358448] Mitchell J (2016c) Dr. James Mitchell talks Enhanced Interrogation techniques. The Hannity Show. Available at: https://video.foxnews.com/v/5228960227001#sp=show-clips (accessed 5 February 2022).

[bibr25-0957154X251358448] Mitchell J (2017a) Plaintiffs V. James Elmer Mitchell and John ‘Bruce’ Jessen’. United States District Court for the Eastern District of Washington at Spokane. Available at: https://www.youtube.com/watch?v=J-J4FbTtXRY&ab_channel=KODXSeattle

[bibr26-0957154X251358448] Mitchell J (2017b) Ex-CIA interrogator on enhanced interrogation. BBC News. Available at: https://www.youtube.com/watch?v=ZdQTm6eQTU8 (accessed 3 February 2022).

[bibr27-0957154X251358448] Mitchell J (2017c) James Mitchell at AFIO Luncheon. AFIO. Available at: https://www.youtube.com/watch?v=E5sRQbvvy4U (accessed 4 February 2022).

[bibr28-0957154X251358448] Mitchell J (2017d) The Mark Steyn show with James Mitchell. The Mark Steyn Show. Available at: https://www.youtube.com/watch?v=3LZA3_1LbWY&ab_channel=MarkSteyn (accessed 2 February 2022).

[bibr29-0957154X251358448] Mitchell J (2018) Dr. James Mitchell. Mike Ritland Show. Available at: https://www.youtube.com/watch?v=8o9IsvIX58Y&ab_channel=MikeRitland (accessed 15 February 2022).

[bibr30-0957154X251358448] Physicians for Human Rights (2014) Doing Harm: Health Professionals’ Central Role in the CIA Torture Program. Physicians for Human Rights. Available at: https://s3.amazonaws.com/PHR_Reports/doing-harm-health-professionals-central-role-in-the-cia-torture-program.pdf (accessed 21 February 2022).

[bibr31-0957154X251358448] Physicians for Human Rights (2015) Lawsuit Filed Against CIA Torture Psychologists. Physicians for Human Rights. Available at: https://phr.org/news/lawsuit-filed-against-cia-torture-psychologists/ (accessed 14 February 2022).

[bibr32-0957154X251358448] Physicians for Human Rights (2020) James Mitchells Testimony at Guantanamo Highlights his Role in US Torture, Debasement of Psychological Ethics. Physicians for Human Rights. Available at: https://phr.org/news/enhanced-interrogation-architect-dr-james-mitchells-testimony-at-guantanamo-highlights-his-role-in-u-s-torture-debasement-of-psychological-ethics-phr/#:~:text=James%20Mitchell%20and%20his%20collaborator,medical%20ethics%20in%20U.S.%20history (accessed 26 February 2022).

[bibr33-0957154X251358448] Physicians for Human Rights (2021) The U.S. Torture Program in the War on Terror. Physicians for Human Rights. Available at: https://phr.org/our-work/resources/twenty-years-of-the-u-s-torture-regime/ (accessed 14 March 2022).

[bibr34-0957154X251358448] Pluta A Zimmerman P (2006) Nuclear terrorism: A disheartening dissent. Survival 48: 55–69.

[bibr35-0957154X251358448] Risen J (2016) After torture, ex-detainee is still captive of ‘the darkness’. Nytimes. Available at: https://www.nytimes.com/2016/10/12/world/cia-torture-abuses-detainee.html (accessed 10 February 2022).

[bibr36-0957154X251358448] Risen J Apuzzo M (2014) CIA, on path to torture, chose haste over analysis. Nytimes. Available at: https://www.nytimes.com/2014/12/16/us/politics/cia-on-path-to-torture-chose-haste-over-analysis-.html (accessed 12 February 2022).

[bibr37-0957154X251358448] Rosenberg C (2020a) Architect of CIA interrogation program testifies at Guantanamo Bay. Nytimes. Available at: https://www.nytimes.com/2020/01/21/us/politics/guantanamo-bay-interrogation.html (accessed 17 January 2022).

[bibr38-0957154X251358448] Rosenberg C (2020b) Chains, shackles and threats: Testimony on torture takes a dramatic turn. Nytimes. Available at: https://nyti.ms/2GtT6Pf (accessed 26 February 2022).

[bibr39-0957154X251358448] Rosenberg C (2020c) He Waterboarded a detainee. Then he had to get the CIA to let him stop. Nytimes. Available at: https://nyti.ms/38qJDUK (accessed 3 January 2022).

[bibr40-0957154X251358448] Rubenstein L (2020) Accountability for medical participation in torture. The Lancet 395: 1827.

[bibr41-0957154X251358448] Sammet K (2006) Avoiding violence by technologies? Rectal feeding in German psychiatry, 1860–85. History of Psychiatry 17: 259–278. 17214428 10.1177/0957154X06061984

[bibr42-0957154X251358448] Senate Select Intelligence Report (SSIR) (2014) Executive Summary. Available at: https://www.documentcloud.org/documents/1377107-sscistudy1.html (accessed 22 January 2020)

[bibr43-0957154X251358448] Shane S (2009) 2 U.S. architects of harsh tactics in 9/11’s wake. Nytimes. Available at: https://www.nytimes.com/2009/08/12/us/12psychs.html (accessed 1 March 2022).

[bibr44-0957154X251358448] Soldz S (2018) It is time for psychologists to face the truth. Psychoanalysis, Self and Context 13(4): 350–367.

[bibr45-0957154X251358448] Stevenson J (2015) Exceptional abhorrence. Survival 57(1): 177–188.

[bibr46-0957154X251358448] Taguba A Cooper S (2017) Tortured souls: The crimes of the war on terrorism. Foreign Affairs 96(3): 116–120.

[bibr47-0957154X251358448] Taub B (2019) How the War on Terror is being written. New Yorker. Available at: https://www.newyorker.com/news/news-desk/how-the-war-on-terror-is-being-written (accessed 10 April 2025).

[bibr48-0957154X251358448] Tayler L Epstein E (2022) Legacy of the Dark Side: The Costs of Unlawful US Detentions and Interrogations Post-9/11. Human Rights Watch. Available at: https://www.hrw.org/news/2022/01/09/legacy-dark-side#_ftn1 (accessed 12 March 2022).

[bibr49-0957154X251358448] Welch M (2009) American pain-ology in the War on Terror: A critique of scientific torture. Theoretical Criminology 13(4): 451–474. DOI: 10.1177/1362480609340394.

[bibr50-0957154X251358448] Welch M (2017) Doing special things to special people in special places: Psychologists in the CIA torture program. The Prison Journal 97(6): 729–749. DOI: 10.1177/0032885517734507.

[bibr51-0957154X251358448] Zubaydah A (2022) How CIA torture pushed me to the edge of death. The Guardian. Available at: https://www.theguardian.com/us-news/2022/jan/29/abu-zubaydah-cia-torture-waterboarding-guantanamo (accessed 17 February 2022).

